# Meta-analysis of human lung cancer microRNA expression profiling studies comparing cancer tissues with normal tissues

**DOI:** 10.1186/1756-9966-31-54

**Published:** 2012-06-06

**Authors:** Peng Guan, Zhihua Yin, Xuelian Li, Wei Wu, Baosen Zhou

**Affiliations:** 1 Department of Epidemiology, School of Public Health, China Medical University, Shenyang 110001, China; 2 Key Laboratory of Cancer Etiology and Prevention (China Medical University), Liaoning Provincial Department of Education, Liaoning, China

**Keywords:** MicroRNAs, Profiling, Lung cancer, Meta-analysis

## Abstract

**Background:**

Lung cancer is the major cause of cancer death globally, it is often diagnosed at an advanced stage and has one of the lowest survival rates of any type of cancer. The common interest in the field of lung cancer research is the identification of biomarkers for early diagnosis and accurate prognosis. There is increasing evidence to suggest that microRNAs play important and complex roles in lung cancer.

**Methods:**

A meta-analysis was conducted to review the published microRNA expression profiling studies that compared the microRNAs expression profiles in lung cancer tissues with those in normal lung tissues. A vote-counting strategy that considers the total number of studies reporting its differential expression, the total number of tissue samples used in the studies and the average fold change was employed.

**Results:**

A total of 184 differentially expressed microRNAs were reported in the fourteen microRNA expression profiling studies that compared lung cancer tissues with normal tissues, with 61 microRNAs were reported in at least two studies. In the panel of consistently reported up-regulated microRNAs, miR-210 was reported in nine studies and miR-21 was reported in seven studies. In the consistently reported down-regulated microRNAs, miR-126 was reported in ten studies and miR-30a was reported in eight studies. Four up-regulated microRNAs (miR-210, miR-21, miR-31 and miR-182) and two down-regulated mcroiRNAs (miR-126 and miR-145) were consistently reported both in squamous carcinoma and adenocarcinoma-based subgroup analysis, with the other 14 microRNAs solely reported in one or the other subset.

**Conclusions:**

In conclusion, the top two most consistently reported up-regulated microRNAs were miR-210 and miR-21. The results of this meta-analysis of human lung cancer microRNA expression profiling studies might provide some clues of the potential biomarkers in lung cancer. Further mechanistic and external validation studies are needed for their clinical significance and role in the development of lung cancer.

## Background

Lung cancer is the leading cause of cancer death in males and the second leading cause of cancer death among females in 2008 globally [1,2]. Lung cancer is often diagnosed at an advanced stage and has one of the lowest survival rates of any type of cancer [3,4]. The common interest in the field of lung cancer research is the identification of biomarkers for early diagnosis and accurate prognosis [5,6], and the general starting point is to compare the gene expression profiles between lung cancer tissues and noncancerous/normal lung tissues. Although many efforts to develop a robust genomic model have been made in this area, controversy exists for their clinical application [7].

Recently, there is increasing evidence to suggest that microRNAs (miRNAs) play important and complex roles in human cancers, including lung cancer [8-10]. miRNAs are a class of small, noncoding, highly stable RNAs that regulate mRNA and protein expression. Several studies have indicated that miRNAs have been involved in regulating various biological processes, such as cellular differentiation, proliferation, angiogenesis, metabolism and cancer development [11-13]. Microarray-based miRNA profiling assays attracted more attention because they constitute the efficient methodology to screen in parallel for the expression of hundreds of miRNAs through extensive sample collections. With the aim at identifying new biomarkers of lung cancer, many investigators have carried out miRNAs expression profiling studies in cell lines, tissue samples or serum samples [9,14,15]. Typically, dozens of miRNAs are identified to be differentially expressed, miRNAs can be either over- or under-expressed, depending on their target downstream genes. Given the fact of a large number of candidate signatures, a logical approach to identify important expression signatures is to search for the intersection of those signatures identified in multiple independent studies [16]. The challenges are how to collate the results of those miRNAs expression profiling studies, when they employed different profiling platforms, and made use of different methods to ascertain differential expression, for example, normalization or significance thresholds. To address these challenges, Griffith and Chan proposed a vote-counting strategy to identify consistent markers when raw data are unavailable [17,18], which gave us insights into the meta-analysis of lung cancer miRNA expression profiling studies.

The starting point of this meta-analysis is to collect those published miRNAs expression profiling studies that compared the miRNAs expression profiles in lung cancer tissues with those in noncancerous/normal lung tissues. Then, the above mentioned vote-counting strategy that considers the total number of studies reporting its differential expression, the total number of tissue samples used in the studies and the average fold change will be employed. The consistently reported differentially miRNAs will be presented and we will also rank the differentially expressed up-regulated and down-regulated miRNAs.

## Methods

### Study selection

PubMed was used to search for lung cancer miRNA expression profiling studies published from January 2003 and May 2012 (last accessed on 15 May 2012), by means of the MeSH terms: ‘lung neoplasms’ and ‘microRNAs’ in combination with the keyword ‘profiling’ and ‘humans’. Eligible studies had to meet the following criteria: (*i*), they were miRNA expression profiling studies in lung cancer patients; (*ii*), they used tissue samples obtained from surgically resected lung tumor and corresponding noncancerous or normal tissues for comparison; (*iii*), use of miRNA microarray methods; (*iv*), reporting of cut-off criteria of differentially expressed miRNAs, and (*v*), validation method and validation sample set reported. Therefore, the miRNA profiling studies using the serum, or sputum samples of lung cancer patients or lung cancer cell lines, or using different miRNA technologies were excluded. Review articles and the studies comparing miRNA expression profiles in lung squamous cell carcinoma from those in lung adenocarcinoma were also excluded.

### Data abstraction

Two investigators (PG and ZY) independently evaluated and extracted the data with the standard protocol and with all the discrepancies resolved by a third investigator (BZ). From the full text and corresponding supplement information, the following eligibility items were collected and recorded for each study: author, journal and year of publication, location of study, selection and characteristics of recruited lung cancer patients, platform of miRNA expression profiling, author defined cut-off criteria of statistically differentially expressed miRNAs and the list of up- and down-regulated miRNA features, and their corresponding fold change (if available).

### Ranking

Each included studies comparing miRNA expression between lung cancer tissues and neighbouring noncancerous or normal lung tissues provided a list of differentially expressed miRNAs. Then, the following vote-counting strategy based method of ranking potential molecular biomarkers, by Griffith [17] and Chan [18], was adopted in the meta-analysis. The differentially expressed miRNAs reported by each study were ranked according to the following order of importance (*i*), number of the studies that consistently reported the miRNA as differentially expressed and with a consistent direction of change; (*ii*), total number of samples for comparison in agreement; (*iii*), average fold change reported by the studies in agreement (only based on the subset of studies with available fold change information). All the comparisons were stepwise made with the online bioinformatics tool (http://jura.wi.mit.edu/bioc/tools/compare.php), and the ranking was performed by Statistical Product and Service Solutions (SPSS 12.0 for windows, SPSS Inc., Chicago, IL, USA).

## Results and discussion

### Included independent studies

A total of 137 relevant publications were indexed in PubMed. According to the inclusion criteria and identification of duplicate publication, only 14 independent studies [19-32] were included in the analysis. The characteristics of these studies are listed in Table 1 in alphabetical order of the first author. Among the fourteen included studies, four studies focused on lung squamous cell carcinoma, three studies focused on lung adenocarcinoma, six studies were about non-small cell lung cancer, and one study based on non-specified lung cancer patients (Table 1). Reference 30 also provided the differentially expression miRNAs by histological type, and the miRNA profiles in lung squamous cell carcinoma of reference 21 was described in a separate publication [33], which made it possible to further explore and compare the deregulated miRNAs in different histological type of lung cancer. Different platforms and various statistical and bio-computational analyses have been utilized in the collected profiling studies. The number of differential miRNAs ranges from 1 to 60, with the median 20. There is one study [19] that only provided the top ten miRNAs of the identified 56 significantly differentially expressed miRNAs. Ten of the fourteen eligible studies provided fold change (FC) information of differentially expressed miRNAs. As one environmental well-known risk factor of lung cancer is tobacco smoking, six studies provided the information of patients’ smoking status. Among them, all the lung cancer patients in reference 19 were current or former heavy smokers and all the lung cancer patients in reference 22 and 25 were never smokers.

**Table 1:** **Fourteen microarray-based human lung cancer microRNA expression profiling studies (lung cancer tissue *versus *normal)**

**First author (reference)**	**Year**	**Lung cancer patients**					**Differentially expressed miRNAs**				
		**Origin**	**Period**	**Cancer type**	**Clinical Stage**	**No. of collected tissue samples (cancer/normal)**	**Platform**	**Cut-off criteria**	**Total**	**Up-regulated features**	**Down-regulated features**
Boeri [19]	2011	Italy	Jun 2000 to Jun 2006	Lung cancer	Satge I to IV (Stage I 60%)	52 (28/24)	The miRNA microarray (Ohio State University, version 2.0)	*P* < 0.001	56	6^†^	4^†^
Dacic [20]	2010	USA (Pittsburgh)	NR	ADC	NR	12 (6/6)	FlexmiR human microRNA pool (Version 8, Exiqon, Vedbaek, Denmark)	FC > 20	7	4	3
Gao [21]	2010	China (Jiangsu, First Affiliated Hospital of Nanjing Medical University)	Apr 2008 to Sep 2008	NSCLC	NR	16 (8/8)	miRCURY™ LNA microRNA Arrays (version 10.0, Exiqon, Vedbaek, Denmark)	FC > 2, *P* < 0.05	27	9	18
			Apr 2008	SCC [Ref 33]	NR	8 (4/4)		FC > 2	31	7	23
Jang [22]	2012	USA (Minnesota)	Jan 1997 to Sep 2008	ADC	Stage I to IV (Stage I 68.0%)	206 (103/103)	Illumina MicroRNA Profiling	FC > 1.5, *P* < 0.01, DR < 0.05	20	10	10
Ma [23]	2011	China (Zhejiang)	NR	NSCLC (SCC:3; ADC:3)	Stage I to IV (Stage I 16.7%)	12 (6/6)	Illuminia Technologies “humanMI_V2”	FDR <0.1	1	1	0
Raponi [24]	2009	USA (Michigan)	Oct 1991 to Jul 2002	SCC	Stage I to IV (Stage I 55%)	71 (61/10)	Ambion mirVana Bioarray (version 2.0)	Signal intensity (log_2_) >6 in at least one group	15	13	2
Seike [25]	2009	USA (Baltimore: 15; Minnesota:7); Japan (Hamamatsu: 6)	2000 to 2004	NSCLC (ADC around 78%)	Stage I to IV (Stage I 75%)	56 (28/28)	The miRNA microarray (Ohio State University, version 3.0)	*P* < 0.01, FDR <0.15	18	5	13
Tan [26]	2011	China (Beijing)	2000 to 2002	SCC	NR	68 (34/34)	CapitalBio platform (CapitalBio Corp.)	Significance analysis of microarray	22	12	10
Võsa [27]	2011	Estonia (Tartu)	2002 to 2008	NSCLC (SCC:18; ADC:20)	Stage I/II (Stage I 92%)	65 (38/27)	Illumina MicroRNA Profiling BeadChip	FC > 2, *P* < 0.01	60	31	29
Wang [28]	2011	China (Jiangsu, Nanjing Chest Hospital)	2006 to 2008	NSCLC (SCC:7; ADC:16)	NR	46 (23/23)	μParaflo microfluidic chip technology (Atactic Technologies, Houston, TX, USA)	FC > 5, *P* < 0.01	40	27	13
Xing [29]	2010	USA (Baltimore)	Mar 2000 to Jun 2003	SCC	Stage I	30 (15/15)	GeneChipR miRNA Array (Affymetrix, Santa Clara, CA, USA)	FC > 1.5, *P* < 0.01	25	7	18
Yanaihara [30]	2006	USA (Baltimore)	1990 to 1999	NSCLC (SCC:39; ADC:65,)	Stage I to IV (Stage I 62.5%)	208 (104/104)	The miRNA microarray Chip (TJU version 1.1)	*P* < 0.001	43	15	28
				SCC		78 (39/39)			16	10	6
				ADC		130 (65/65)			17	5	12
Yang [31]	2010	China (Shaanxi)	NR	SCC	NR	6 (3/3)	miRCURY™ LNA array (version 10.0, Exiqon, Vedbaek, Denmark)	FC > 1.5, *P* < 0.05	9	2	7
Yu [32]	2010	USA (Baltimore)	NR	ADC	Stage I	40 (20/20)	Taqman human miRNA array A (System Biosciences, Mountain View, CA)	FC > 1.5, *P* < 0.01	20	11	9

*Abbreviations:* ADC, adenocarcinoma/adenosquamous carcinoma; FC, fold change; FDR, false discovery rate; miRNAs, microRNAs; NR, not reported; NSCLC, non-small cell lung cancer; SCC, squamous cell carcinoma.

^†^ Only the top ten miRNAs of the identified 56 significantly differentially expressed miRNAs were provided.

### Differentially expressed miRNAs

A total of 184 differentially expressed miRNAs were reported in the fourteen miRNA expression profiling studies that compared lung cancer tissues with normal tissues, with 61 miRNAs (33.2%) were reported in at least two studies. Among the 61 differentially expressed miRNAs, 54 miRNAs (88.5%) were with a consistent direction, 26 were reported to be up-gulated (Table 2) and 28 down-regulated (Table 3). The seven inconsistently reported miRNAs are listed in Table 4.

**Table 2:** **Consistently reported up-regulated miRNAs (*n*** **= 26) in profiling studies (lung cancer tissue *versus *normal)**

**miRNA name**^**a**^	**No. of studies with same direction (reference)**	**No. of tissue samples tested**	**Subset of studies with fold change**			
			**No. of studies**	**No. of tissue samples tested**	**Mean fold change**	**Range**
miR-210	9 (19,22,24,25,26,27,29,30,32)	796	6	449	2.65	1.51 - 5.10
miR-21	7 (19,21,25,28,29,30,32)	448	6	240	4.39	1.74 - 13.60
miR-182	6 (22,24,26,27,28,32)	496	4	357	6.34	1.85 - 19.00
miR-31	6 (21,22,26,27,29,32)	425	5	357	2.89	1.58 - 4.80
miR-205	5 (26,27,28,29,30)	417	3	141	23.20	2.99 - 54.30
miR-200b	5 (19,25,26,28,32)	262	4	194	3.69	1.30 - 9.80
miR-183	4 (22,24,27,28)	388	3	317	5.94	2.11 - 11.60
miR-203	3 (24,26,30)	347	0	-	-	-
miR-196a	3 (22,27,28)	317	3	317	37.50	2.10 - 101.80
miR-708	3 (22,27,29)	301	3	301	3.20	1.85 - 5.50
miR-92b	3 (27,28,32)	151	3	151	3.71	1.54 - 6.80
miR-193b	3 (21,26,27)	149	2	81	4.68	2.56 - 6.80
miR-106a	2 (24,30)	279	0	-	-	-
miR-21*	2 (22,27)	271	2	271	2.23	2.16 - 2.30
miR-135b	2 (21,22)	222	2	222	2.29	2.28 - 2.31
miR-96	2 (22,23)	218	2	218	171.56	2.30 - 340.81
miR-17-5p	2 (24,27)	136	1	65	3.80	-
miR-20b	2 (24,28)	117	1	46	5.70	-
miR-18a	2 (26,28)	114	1	46	7.80	-
miR-200a	2 (24,32)	111	1	40	1.86	-
miR-93	2 (24,32)	111	1	40	1.68	-
miR-130b	2 (26,32)	108	1	40	1.57	-
miR-200c	2 (24,29)	101	1	30	1.66	-
miR-375	2 (28,32)	86	2	86	5.35	2.89 - 7.80
miR-20a	2 (20,24)	83	0	-	-	-
miR-18b	2 (20,26)	80	0	-	-	-

^a^ The asterisk is part of the miRNA nomenclature system and is not linked to any footnote specific to this table.

**Table 3:** **Consistently reported down-regulated miRNAs (*n*** **= 28) in profiling studies (lung cancer tissue *versus *normal)**

**miRNA name**^**a**^	**No. of studies with same direction (reference)**	**Total number of tissue samples tested**	**Subset of studies with fold change**			
			**No. of studies**	**Total number of tissue samples tested**	**Mean fold change**	**Range**
miR-126	10 (19,21,25,26,27, 28,29,30,31,32)	587	8	311	0.33	0.00 - 0.69
miR-30a	8 (19,21,25,26,27,28,29,31)	339	7	271	0.36	0.04 - 0.61
miR-451	6 (19,21,25,27,28,29)	265	6	265	0.37	0.01 - 0.53
miR-486-5p	5 (19,22,26,27,28)	437	4	369	0.39	0.13 - 0.53
miR-30d	5 (21,25,28,29,31)	154	5	154	0.34	0.08 - 0.57
miR-145	4 (26,28,30,32)	362	2	86	0.23	0.09 - 0.38
miR-143	4 (21,28,30,32)	310	3	102	0.33	0.13 - 0.59
miR-139-5p	3 (22,27,29)	301	3	301	0.55	0.40 - 0.64
miR-126*	3 (21,25,30)	280	2	72	0.33	0.20 - 0.45
miR-140-3p	3 (26,27,28)	179	2	111	0.29	0.17 - 0.42
miR-138	3 (25,26,32)	164	2	96	0.64	0.56 - 0.72
miR-30b	3 (25,28,29)	132	3	132	0.41	0.11 - 0.58
miR-486	3 (25,29,32)	126	3	126	0.44	0.34 - 0.53
miR-101	3 (21,27,31)	87	3	87	0.34	0.24 - 0.48
miR-125a	2 (24,30)	279	0	-	-	-
miR-198	2 (27,30)	273	1	65	0.25	-
miR-144*	2 (22,27)	271	2	271	0.31	0.14 - 0.48
miR-140	2 (30,32)	248	1	40	0.66	-
miR-218	2 (22,32)	246	2	246	0.61	0.60 - 0.62
miR-32	2 (20,30)	220	0	-	-	-
miR-338-3p	2 (26,27)	133	1	65	0.20	-
miR-99a	2 (27,28)	111	2	111	0.31	0.20 - 0.42
miR-195	2 (26,29)	98	1	30	0.53	-
miR-497	2 (26,29)	98	1	30	0.66	-
miR-30c	2 (25,29)	86	2	86	0.58	0.54 - 0.61
miR-130a	2 (21,27)	81	2	81	0.46	0.45 - 0.46
miR-16	2 (28,29)	76	2	76	0.37	0.18 - 0.57
miR-139	2 (29,32)	70	2	70	0.53	0.49 - 0.58

^a^ The asterisk is part of the miRNA nomenclature system and is not linked to any footnote specific to this table.

**Table 4:** **Inconsistently reported miRNAs (*n*** **= 7) in profiling studies (lung cancer tissue *versus *normal)**

**miRNA name**^**a**^	**Direction of expression**	**Reference**	**Total number of tissue samples tested**	**Mean fold change**
miR-224	↑	24,27	136	3.4
	↓	30	208	-
miR-9	↑	22,27	271	8.59
	↓	30	208	-
miR-150	↑	30	208	-
	↓	28,32	86	0.38
miR-219-1	↑	19	52	1.6
	↓	30	208	-
miR-125a-5p	↑	31	6	1.56
	↓	26,29	98	0.62
miR-429	↑	26	68	-
	↓	29	30	0.50
miR-24-2*	↑	21	16	2.33
	↓	27	65	0.5

^a^ The asterisk is part of the miRNA nomenclature system and is not linked to any footnote specific to this table.

In the panel of consistently reported up-regulated miRNAs, miR-210 was reported in nine studies (average FC: 2.65) and miR-21 was reported in seven studies (average FC: 4.39). In the consistently reported down-regulated miRNAs, miR-126 was reported in ten studies (average FC: 0.33), and miR-30a was reported in eight studies (average FC: 0.36).

Subgroup analysis on histological type was conducted for further comparison. In the six studies based on the tissues from lung squamous carcinoma patients [24,26,29-31,33], nineteen deregulated miRNAs were consistently reported in at least two studies (8 up-regulated and 11 down-regulated) with miR-210 as the most frequent reported up-regulated miRNA (Table 5). In the subset of four studies about lung adenocarcinoma [20,22,30,32], seven miRNAs were consistently reported, with miR-210 as the most frequent reported up-regulated miRNA (Table 6). Four up-regulated miRNAs (miR-210, miR-21. miR-31 and miR-182) and two down-regulated miRNAs (miR-126 and miR-145) were consistently reported both in squamous carcinoma and adenocarcinoma-based analysis, with the other 14 miRNAs solely reported in one subset or the other (Tables 5 and 6).

**Table 5:** **Deregulated miRNAs (*n*** **= 19) consistently reported in profiling studies (lung SCC tissue *versus *normal)**

**Direction of expression**	**miRNA name**^**a**^	**No. of studies with same direction (reference)**	**Total number of tissue samples tested**	**Subset of studies with fold change**			
				**No. of studies**	**Total number of tissue samples tested**	**Mean fold change**	**Range**
Up-regulated	miR-210	4 (24,26,29,30)	247	1	30	2.37	-
	miR-203	3 (24,26,30)	217	0	-	-	-
	miR-205	3 (26,29,30)	176	1	30	2.99	-
	miR-21	3 (29,30,33)	116	2	38	2.53	1.74 - 3.31
	miR-31	3 (26,29,33)	106	2	38	4.42	1.58 - 7.26
	miR-182	2 (24,26)	139	0	-	-	-
	miR-200c	2 (24,29)	101	1	30	1.66	-
	miR-18a	2 (26,33)	76	1	8	2.24	-
Down-regulated	miR-126	4 (26,29,31,33)	112	3	44	0.18	0.00 - 0.42
	miR-30a	4 (26,29,31,33)	112	3	44	0.28	0.11 - 0.53
	miR-30d	3 (29,31,33)	44	3	44	0.33	0.22 - 0.54
	miR-195	2 (26,29)	98	1	30	0.53	-
	miR-497	2 (26,29)	98	1	30	0.66	-
	miR-126*	2 (30,33)	86	1	8	0.16	-
	miR-143	2 (30,33)	86	1	8	0.24	-
	miR-145	2 (26,33)	76	1	8	0.48	-
	miR-451	2 (29,33)	38	2	38	0.37	0.22 - 0.53
	miR-30b	2 (29,33)	38	2	38	0.50	0.48 - 0.53
	miR-101	2 (31,33)	14	2	14	0.34	0.29 - 0.39

^a^ The asterisk is part of the miRNA nomenclature system and is not linked to any footnote specific to this table. SCC, squamous cell carcinoma.

**Table 6:** **Deregulated miRNAs (*n*** **= 7) consistently reported in profiling studies (lung ADC tissue *versus *normal)**

**Direction of expression**	**miRNA name**	**No. of studies with same direction (reference)**	**Total number of tissue samples tested**	**Subset of studies with fold change**			
				**No. of studies**	**Total number of tissue samples tested**	**Mean fold change**	**Range**
Up-regulated	miR-210	3 (22,30,32)	376	2	246	1.96	1.75 - 2.17
	miR-182	2 (22,32)	246	2	246	2.03	1.85 - 2.22
	miR-31	2 (22,32)	246	2	246	1.83	1.60 - 2.05
	miR-21	2 (30,32)	170	1	40	2.56	-
Down-regulated	miR-218	2 (22,32)	246	2	246	0.61	0.60 - 0.62
	miR-145	2 (30,32)	170	1	40	0.38	-
	miR-126	2 (30,32)	170	1	40	0.46	-

ADC, adenocarcinoma/adenosquamous carcinoma.

### Factors to consider for miRNAs as biomarkers

To our knowledge, no meta-analysis of miRNA profiling studies has investigated lung cancer specially. This kind of systematic review has been proved to be useful in exploring candidate miRNA biomarkers in human colorectal cancer [34]. The present study suggested several promising miRNAs that have been consistently reported with average more than 2-fold change. Their potential targets may provide a clue to the role of miRNAs in tumorigenesis and the underlying mechanisms.

There are several factors needed to be considered when choosing miRNAs as candidate clinical biomarkers of lung cancer. First, the biological complexities should be well understood. A single miRNA may have many targets, and also, a specific mRNA may be regulated by multiple different miRNAs [35]. More understanding of molecular mechanisms that can mediate miRNA dysregulations and the targets of the miRNAs would advance their use in clinical settings.

Second, there should be sufficient information about their pattern of expression in different kinds of specimens in target populations. The release mechanism of miRNAs can be via tumor-derived microvesicles or exosomes [36,37]. It has been indicated that circulating miRNAs in plasma could be more tissue-specific than tumor-specific [8,38], thus our study focused on the profiling studies that compared miRNA profiles in lung cancer tissues with those in normal lung tissues. Boeri and colleagues found that miRNAs deregulated in tissue specimens were rarely detected in plasma samples, which further strengthened the high tissue-specificity of miRNAs and suggested the predictive role of plasma miRNAs independent from tissue specimens [19]. In the context of the inconsistent profiles between tissue-based and plasma-based result, however, some consistently reported miRNAs in tissue-based profiling studies, for example, a panel of miR-21, miR-210 and miR-486-5p, have been validated in plasma-based studies to confirm their diagnostic value in the diagnosis of lung cancer with solitary pulmonary nodules [39]. Future studies that based on parallel plasma and tissue samples may provide more solid evidence. For the included profiling studies in which adjacent corresponding normal lung tissue served as an expression baseline, we need to know that adjacent appearing morphologically normal tissue may contain molecular changes associated with cancer [40,41].

Third, rigorous validation and demonstration of reproducibility in an independent population are necessary to confirm the predictive value of miRNAs. One of the most frequently investigated miRNAs is miR-21, it ranks second among consistently reported up-regulated miRNAs in this meta-analysis, it has been also reported to be associated with prognosis in several kinds of cancer [42-44]. From the prognostic point of view, over expression of miR-21 has been reported to be independently associated with reduced survival of pancreatic ductal adenocarcinoma [43]. High miR-21 expression was also associated with poor survival of colon adenocarcinoma in both the training cohort (US test cohort of 84 patients with incident colon adenocarcinoma, recruited between 1993 and 2002) and validation cohort (independent Chinese cohort of 113 patients recruited between 1991 and 2000) [44]. However, when expression of miR-21, miR-29b, miR-34a/b/c, miR-155, and let-7a was determined by quantitative real-time PCR in formalin-fixed paraffin-embedded tumor specimens from 639 patients who participated in the International Adjuvant Lung Cancer Trial (IALT), there was a deleterious borderline prognostic effect of lowered miR-21 expression [45].

## Conclusions

In conclusion, the top two most consistently reported up-regulated miRNAs were miR-210 and miR-21. The results of this meta-analysis of human lung cancer miRNA expression profiling studies might provide some clues of the potential biomarkers in lung cancer. Further mechanistic and external validation studies are needed for their clinical significance and role in the development of lung cancer.

## Abbreviations

ADC: Adenocarcinoma/adenosquamous carcinoma; FC: Fold change; FDR: False discovery rate; miRNAs: MicroRNAs; NR: Not reported; NSCLC: Non-small cell lung cancer; SCC: Squamous cell carcinoma.

## Competing interests

The authors declare that they have no competing interests.

## Authors’ contributions

PG conceived the study and drafted the manuscript. PG and ZY collected and analyzed the data, PG and ZY also secured funding. XL, WW and BZ contributed to the quality control of study inclusion and discussion. All authors read and approved the final manuscript.
